# Detecting and classifying lesions in mammograms with Deep Learning

**DOI:** 10.1038/s41598-018-22437-z

**Published:** 2018-03-15

**Authors:** Dezső Ribli, Anna Horváth, Zsuzsa Unger, Péter Pollner, István Csabai

**Affiliations:** 1 Department of Physics of Complex Systems, Eötvös Loránd University, Budapest, Hungary; 2 3rd Department of Internal Medicine, Semmelweis University, Budapest, Hungary; 3 Department of Radiology, Semmelweis University, Budapest, Hungary; 4 MTA-ELTE Statistical and Biological Physics Research Group, Hungarian Academy of Sciences, Budapest, Hungary

## Abstract

In the last two decades, Computer Aided Detection (CAD) systems were developed to help radiologists analyse screening mammograms, however benefits of current CAD technologies appear to be contradictory, therefore they should be improved to be ultimately considered useful. Since 2012, deep convolutional neural networks (CNN) have been a tremendous success in image recognition, reaching human performance. These methods have greatly surpassed the traditional approaches, which are similar to currently used CAD solutions. Deep CNN-s have the potential to revolutionize medical image analysis. We propose a CAD system based on one of the most successful object detection frameworks, Faster R-CNN. The system detects and classifies malignant or benign lesions on a mammogram without any human intervention. The proposed method sets the state of the art classification performance on the public INbreast database, AUC = 0.95. The approach described here has achieved 2nd place in the Digital Mammography DREAM Challenge with AUC = 0.85. When used as a detector, the system reaches high sensitivity with very few false positive marks per image on the INbreast dataset. Source code, the trained model and an OsiriX plugin are published online at https://github.com/riblidezso/frcnn_cad.

## Introduction

### Screening mammography

Breast cancer is the most common cancer in women and it is the main cause of death from cancer among women in the world^1^. Screening mammography has been shown to reduce breast cancer mortality by 38–48% among participants^2^. In the EU 25 of the 28 member states are planning, piloting or implementing screening programs to diagnose and treat breast cancer in an early stage^3^. During a standard mammographic screening examination, X-ray images are captured from 2 angles of each breast. These images are inspected for malignant lesions by one or two experienced radiologists. Suspicious cases are called back for further diagnostic evaluation.

Screening mammograms are evaluated by human readers. The reading process is monotonous, tiring, lengthy, costly and most importantly, prone to errors. Multiple studies have shown that 20–30% of diagnosed cancers could be found retrospectively on the previous negative screening exam by blinded reviewers^4–9^. The problem of missed cancers still persists despite modern full field digital mammography (FFDM)^4,8^. The sensitivity and specificity of screening mammography is reported to be between 77–87% and 89–97% respectively. These metrics describe the average performance of readers, and there is substantial variance in the performance of individual physicians, with reported false positive rates between 1–29%, and sensitivities between 29–97%^10–12^. Double reading was found to improve the performance of mammographic evaluation and it has been implemented in many countries^13^. Multiple readings can further improve diagnostic performance up to more than 10 readers, proving that there is room for improvement in mammogram evaluation beyond double reading^14^.

### Computer-aided detection in mammographic screening

Computer-aided detection (CAD) solutions were developed to help radiologists in reading mammograms. These programs usually analyse a mammogram and mark the suspicious regions, which should be reviewed by the radiologist^15^. The technology was approved by the FDA and had spread quickly. By 2008, in the US, 74% of all screening mammograms in the Medicare population were interpreted with CAD, however the cost of CAD usage is over $400 million a year^11^.

The benefits of using CAD are controversial. Initially several studies have shown promising results with CAD^6,16–20^. A large clinical trial in the United Kingdom has shown that single reading with CAD assistance has similar performance to double reading^21^. However, in the last decade multiple studies concluded that currently used CAD technologies do not improve the performance of radiologists in everyday practice in the United States^11,22,23^. These controversial results indicate that CAD systems need to be improved before radiologists can ultimately benefit from using the technology in everyday practice.

Currently used CAD approaches are based on describing an X-ray image with meticulously designed hand crafted features, and machine learning for classification on top of these features^15,24–27^. In the field of computer vision, since 2012, deep convolutional neural networks (CNN) have significantly outperformed these traditional methods^28^. Deep CNN-s have reached, or even surpassed, human performance in image classification and object detection^29^. These models have tremendous potential in medical image analysis. Several studies have attempted to apply Deep Learning to analyse mammograms^27,30–32^, but the problem is still far from being solved.

### The Digital Mammography DREAM Challenge

The Digital Mammography DREAM Challenge (DM challenge)^33,34^ asked participants to write algorithms which can predict whether a breast in a screening mammography exam will be diagnosed with cancer. The dataset consisted of 86000 exams, with no pixel level annotation, only a binary label indicating whether breast cancer was diagnosed within the next 12 months after the exam. Each side of the breasts were treated as separate cases that we will call breast-level prediction in this paper. The participants had to upload their programs to a secure cloud platform, and were not able to download or view the images, nor interact with their program during training or testing. The DM challenge provided an excellent opportunity to compare the performance of competing methods in a controlled and fair way instead of self-reported evaluations on different or proprietary datasets.

## Material and Methods

### Data

Mammograms with pixel level annotations were needed to train a lesion detector and test the classification and localisation performance. We trained the model on the public Digital Database for Screening Mammography (DDSM)^35^ and a dataset from the Semmelweis University in Budapest, and tested it on the public INbreast^36^ dataset. The images used for training contain either histologically proven cancers or benign lesions which were recalled for further examinations, but later turned out to be nonmalignant. We expected that training with both kinds of lesions will help our model to find more lesions of interest, and differentiate between malignant and benign examples.

The DDSM dataset contains 2620 digitised film-screen screening mammography exams, with pixel-level ground truth annotation of lesions. Cancerous lesions have histological proof. We have only used the DDSM database for training our model and not evaluating it. The quality of digitised film-screen mammograms is not as good as full field digital mammograms therefore evaluation on these cases is not relevant. We have converted the lossless jpeg images to png format, mapped the pixel values to optical density using calibration functions from the DDSM website, and rescaled the pixel values to the 0–255 range.

The dataset from the Department of Radiology at Semmelweis University in Budapest, Hungary contains 847 FFDM images of 214 exams from 174 patients, recorded with a Hologic LORAD Selenia device. Institutional board approval was obtained for the dataset. This dataset was not available for the full period of the DM challenge, therefore it was only used for improvement in the second stage of the DM challenge, after pixel level annotation by the authors.

The INbreast dataset contains 115 FFDM cases with pixel-level ground truth annotations, and histological proof for cancers^36^. We adapted the INbreast pixel level annotations to suit our testing scenario. We ignored all benign annotations, and converted the malignant lesion annotations to bounding boxes. We excluded 8 exams which had unspecified other findings, artefacts, previous surgeries, or ambiguous pathological outcome. The images had low contrast, therefore we adjusted the window of the pixel levels. The pixel values were clipped to be minimum 500 pixel lower and maximum 800 pixels higher than the mode of the pixel value distribution (excluding the background) and were rescaled to the 0–255 range.

### Data Availability

The DDSM dataset is available online at http://marathon.csee.usf.edu/Mammography/Database.html.

The INBreast dataset can be requested online at http://medicalresearch.inescporto.pt/breastresearch/index.php/Get_INbreast_Database.

The dataset acquired from Semmelweis University (http://semmelweis.hu/radiologia/) was used with a special license therefore is not publicly available, however the authors can supply data upon reasonable request and permission from the University.

### Methods

**Figure 1:**
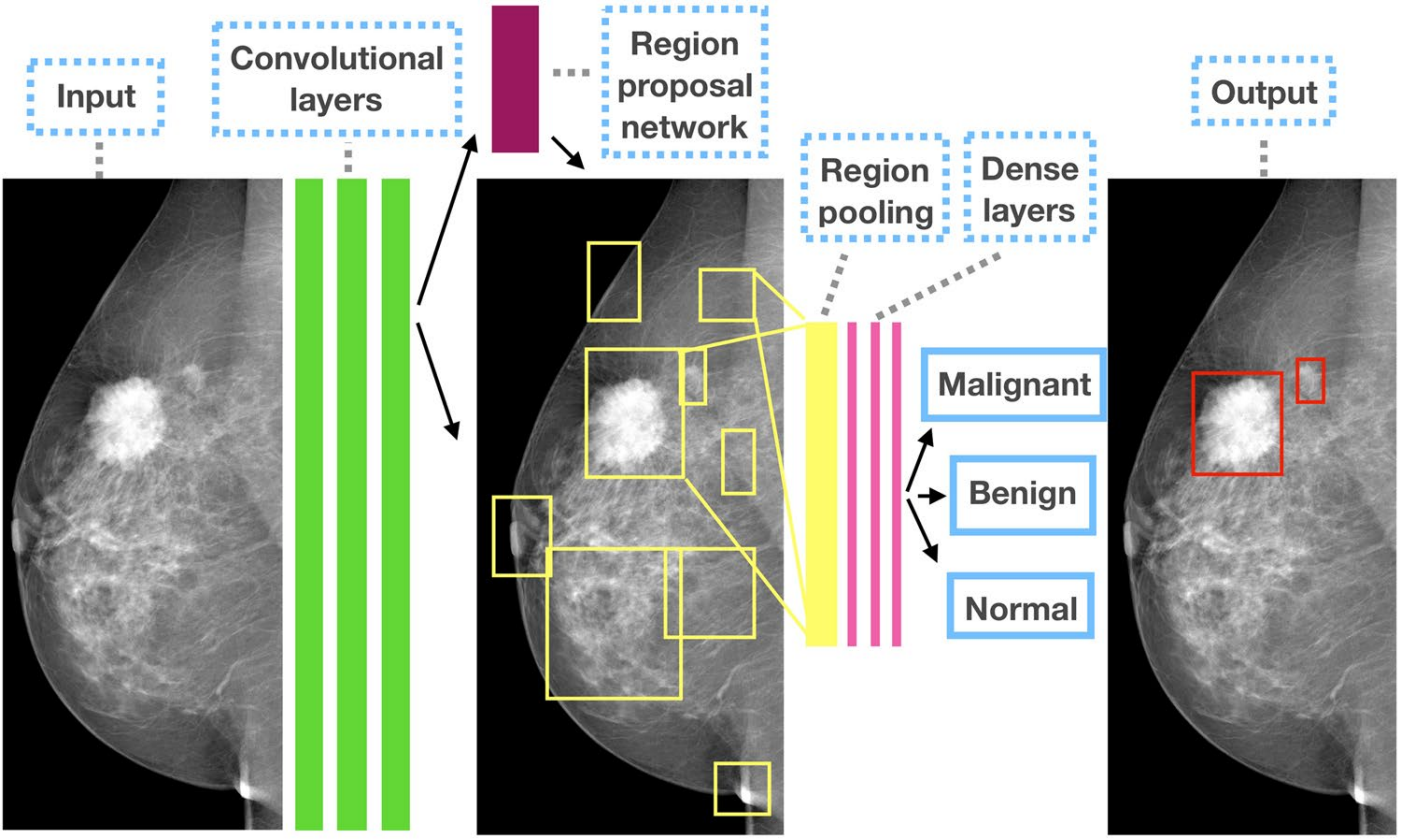
The outline of the Faster R-CNN model for CAD in mammography.

The heart of our model is a state of the art object detection framework, Faster R-CNN^37^. Faster R-CNN is based on a convolutional neural network with additional components for detecting, localising and classifying objects in an image. Faster R-CNN has a branch of convolutional layers, called Region Proposal Network (RPN), on top of the last convolutional layer of the original network, which is trained to detect and localise objects on the image, regardless of the class of the object. It uses default detection boxes with different sizes and aspect ratios in order to find objects with varying sizes and shapes. The highest scoring default boxes are called region proposals for the other branch of the network. The other branch of the neural network evaluates the signal coming from each proposed region of the last convolutional layer, which is resampled to a fix size. Both branches try to solve a classification task to detect the presence of objects, and a bounding-box regression task in order to refine the boundaries of the object present in the region. From the detected overlapping objects, the best predictions are selected using non-maximum suppression. Further details about Faster R-CNN can be found in the original article^37^. An outline of the model can be seen in Fig. 1.

The base CNN used in our model was a VGG16 network, which is a 16 layer deep CNN^38^. The final layer can detect 2 kinds of objects in the images, benign or malignant lesions. The model’s output is a bounding box for each detected lesion, and a score, which reflects the confidence in the class of the lesion. To describe an image with one score, we calculate the maximum of the scores of all malignant lesions detected in the image. For multiple images of the same breast, we take the average of the scores of individual images. For the DM challenge, we trained 2 models using shuffled training datasets. When ensembling these models, the score of an image was the average score of the individual models. This approach was motivated by a previous study on independent human readers, and it has proven reasonably effective, while being both simple and flexible^14^.

We used the framework developed by the authors of Faster R-CNN^37^, which was built in the Caffe framework for deep learning^39^. During training, we optimized both the object detection and classifier part of the model at the same time, which is called joint optimization^37^. We used backpropagation and stochastic gradient descent with weight decay. The initial model used for training was pretrained on 1.2 million images from the ImageNet dataset^38^.

The mammograms were downscaled isotropically so their longer side were smaller than 2100 pixels and their shorter side were smaller than 1700 pixels. This resolution is close to the maximum size that fits in the memory of the graphics card used. The aspect ratio was selected to fit the regular aspect ratio of Hologic images. We have found that higher resolution yields better results as a previous model with images downscaled to 1400 × 1700 pixel resolution had 0.08 lower AUC in the DM challenge. We applied vertical and horizontal flipping to augment the training dataset. Mammograms contain fewer objects than ordinary images and during the initial inspection of the training behaviour we have observed pathologically few positive regions in minibatches. To solve the class balance problem we decreased the Intersection over Union (IoU) threshold for foreground objects in the region proposal network from 0.7 to 0.5. This choice allowed more positive examples in a minibatch, and effectively stabilized training. Relaxation of positive examples is also supported by the fact that lesions on a mammogram have much less well-defined boundaries than a car or a dog on a traditional image. The IoU threshold of the final non-maximum suppression (nms) was set to 0.1, because mammograms represent a compressed, and relatively thin 3D space compared to ordinary images, therefore overlapping detections are expected to occur less often than in usual object detection. The model was trained for 40k iterations, this number was previously found to be close to optimal by testing multiple models on the DM challenge training data. The model was trained and evaluated on an Nvidia GTX 1080Ti graphics card. The results presented in this article using the INbreast dataset were obtained with a single model. Our final entry in the DM challenge was an ensemble of 2 models, which was the maximum number of models we could run, given the runtime restrictions in the challenge.

## Results

### Cancer classification

**Figure 2:**
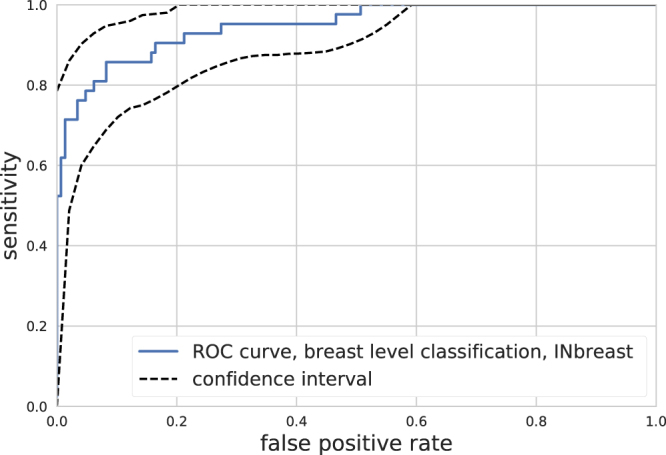
Classification performance. The solid blue line shows the ROC curve on the INbreast dataset on breast level, AUC = 0.95, the dashed lines show the 95 percentile interval of the curve based on 10000 bootstrap samples.

We also evaluated the model’s performance on the public INbreast dataset with the receiver operating characteristics (ROC) metric, Fig. 2. The INbreast dataset contains many exams with only one laterality, therefore we evaluated predictions for each breast. The system achieved AUC = 0.95, (95 percentile interval: 0.91 to 0.98, estimated from 10000 bootstrap samples). This is the highest AUC score reported on the INbreast dataset with a fully automated system based on a single model, to our best knowledge.

### FROC analysis

**Figure 3:**
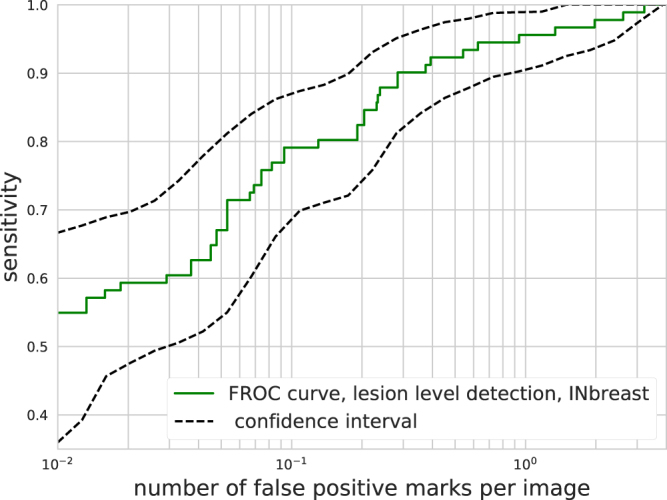
FROC curve on the INbreast dataset. Sensitivity is calculated on a per lesion basis. The solid curve with squares shows the results using all images, while the dashed lines show the 95 percentile interval from 10000 bootstrap samples.

In order to test the model’s ability to detect and accurately localise malignant lesions, we evaluated the predictions on the INbreast dataset using the Free-response ROC (FROC) curve^40^. The FROC curve shows sensitivity (fraction of correctly localised lesions) as a function of the number of false positive marks put on an image Fig. 3.

A detection was considered correct if the center of the proposed lesion fell within a ground truth box. The same criteria is generally used when measuring the performance of currently used CAD products^24,41,42^. The DM challenge dataset has no lesion annotation, therefore we cannot use it for FROC analysis.

As seen in Fig. 3, our model was able to detect malignant lesions with a sensitivity of $0.9$ and $0.3$ false positive marks per image. The reported number of false positive marks per image for commercially available CAD systems ranges from 0.3 to 1.25^11,16–20,41–43^. The lesion based sensitivities of commercially available CAD systems are generally reported to be around 0.75–0.77 for scanned film mammograms^17,19,20^, and $0.85$ for FFDM^42,43^. Our model achieves slightly better detection performance on the INbreast dataset than the reported characteristics of commercial CAD systems, although it is important to note that results obtained on different datasets are not directly comparable.

### Examples

**Figure 4:**
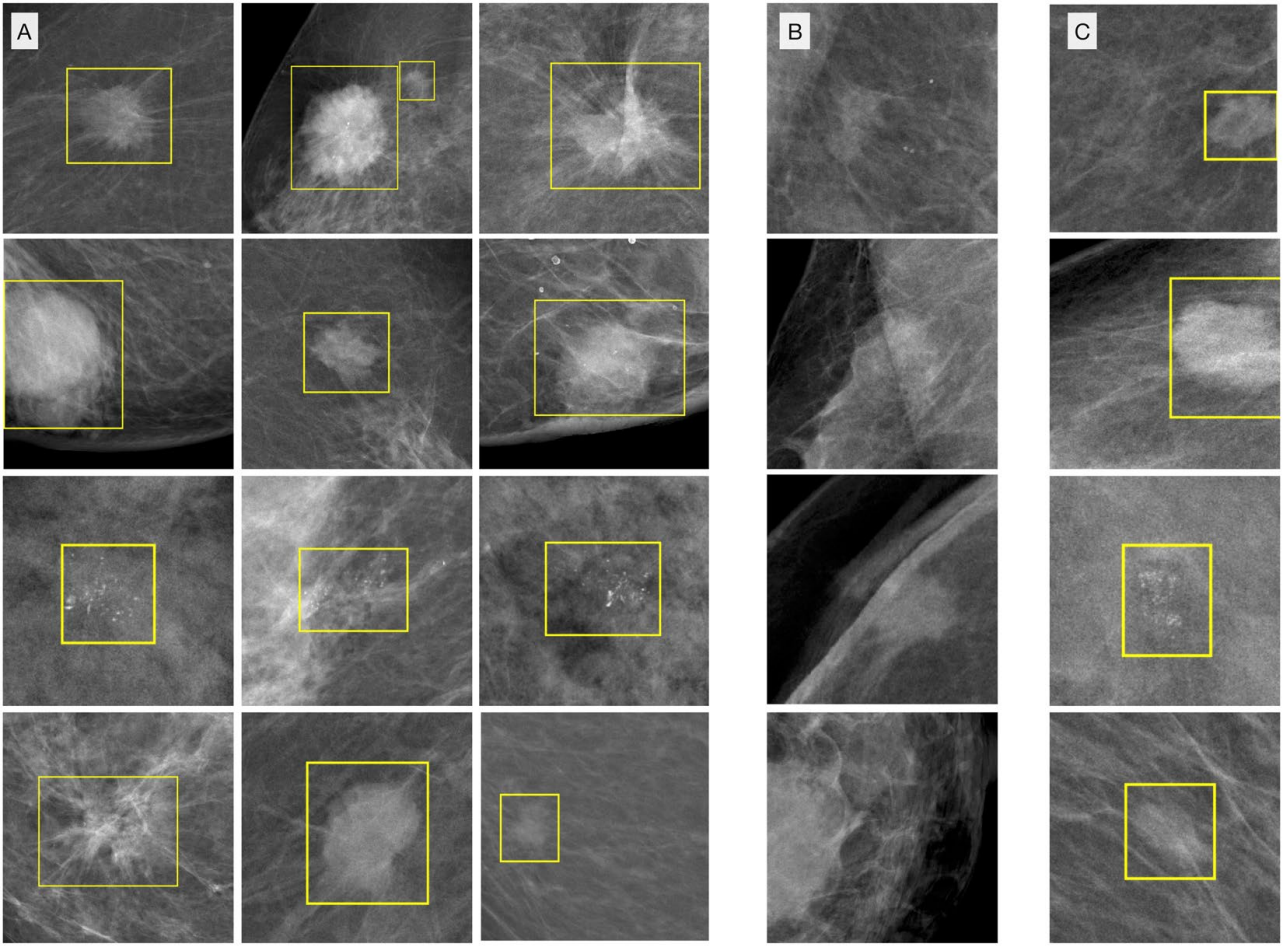
Detection examples: The yellow boxes show the lesion proposed by the model. The threshold for these detections was selected to be at lesion detection sensitivity = $0.9$. (**A**) Correctly detected malignant lesions. (**B**) Missed malignant lesions. (**C**) False positive detections, Courtesy of the Breast Research Group, INESC Porto, Portugal^36^.

To demonstrate the characteristics and errors of the detector, we created a collection of correctly classified, false positive and missed malignant lesions of the INbreast dataset, see in Fig. 4. The score threshold for the examples was defined at a sensitivity of $0.9$ and $0.3$ false positive marks per image.

After inspecting the false positive detections, we found that most were benign masses or calcifications. Some of these benign lesions were biopsy tested according to the case descriptions of the INbreast dataset. While 10% of the ground truth malignant lesions were missed at this detection threshold, these were not completely overlooked by the model. With a score threshold which corresponds to $3$ false positive marks per image, all the lesions were correctly detected (see Fig. 3). Note, that the exact number of false positive and true positive detections slightly varies with different samples of images, indicated by the area in Fig. 3.

## Discussion

We have proposed a Faster R-CNN based CAD approach, which achieved 2nd place in the Digital Mammography DREAM Challenge with an AUC = $0.85$ score on the final validation dataset. The competition results proved that the method described in this article is one of the best approaches for cancer classification in mammograms. Out of the top contenders in the DM challenge, our method was the only one that was based on the detection of malignant lesions. For our approach, whole image classification is just a trivial step from the detection task. We believe that a lesion detector is clinically much more useful than a simple image classifier. A classifier only gives a single score per case or breast, and it is not able to locate the cancer, which is essential for further diagnostic tests or treatment.

We have evaluated the model on the publicly available INbreast dataset. The system is able to detect 90% of the malignant lesions in the INbreast dataset with only 0.3 false positive marks per image. It also sets the state of the art performance in cancer classification on the publicly available INbreast dataset. The system uses the mammograms as the only input without any annotation or user interaction.

An object detection framework developed to detect objects in ordinary images shows excellent performance. This result indicates that lesion detection on mammograms is not very different from a regular object detection task. Therefore the expensive, traditional CAD solutions, which have controversial efficiency, could be replaced with the recently developed, deep learning based, open source object detection methods in the near future. Provided with more training data, these models have the potential to become significantly more accurate. The FROC analysis results suggest that the proposed model could be applied as a perception enhancer tool, which could help radiologists to detect more cancers.

A limitation of our study comes from the small size of the publicly available pixel-level annotated dataset. While the classification performance of the model has been evaluated on a large screening dataset, the detection performance could only be evaluated on the small INbreast dataset.
